# Nomogram based on immune-inflammatory indicators and age-adjusted charlson comorbidity index score to predict prognosis of postoperative parotid gland carcinoma patients

**DOI:** 10.1186/s12903-024-04490-5

**Published:** 2024-06-22

**Authors:** Hao Cheng, Jin-Hong Xu, Jia-Qi He, Chen-Chen Wu, Jia-Fan Li, Xue-Lian Xu

**Affiliations:** 1 Department of Radiotherapy Oncology, The First Affiliated Hospital of Xinxiang Medical University, 88 Jiankang Road, Xinxiang, Henan, 453100 Henan China; 2 Department of Otolaryngology, AnYang District Hospital, Anyang, Henan, 455000 China; 3 Department of Radiotherapy Oncology, Affiliated Cancer Hospital of Zhengzhou University, Zhengzhou, Henan, 450000 China

**Keywords:** Parotid gland carcinoma, Immune-inflammatory-nutrition indicators, Age-adjusted charlson comorbidity index score, Prognosis, Nomogram

## Abstract

**Background:**

Parotid gland carcinoma (PGC) is a rare malignant tumor. The purpose of this study was to investigate the role of immune-inflammatory-nutrition indicators and age-adjusted Charlson comorbidity index score (ACCI) of PGC and develop the nomogram model for predicting prognosis.

**Method:**

All patients diagnosed with PGC in two tertiary hospitals, treated with surgical resection, from March 2012 to June 2018 were obtained. Potential prognostic factors were identified by univariate and multivariate Cox regression analyses. The nomogram models were established based on these identified independent prognostic factors. The performance of the developed prognostic model was estimated by related indexes and plots.

**Result:**

The study population consisted of 344 patients with PGC who underwent surgical resection, 285 patients without smoking (82.8%), and 225 patients (65.4%) with mucoepidermoid carcinoma, with a median age of 50.0 years. American Joint Committee on Cancer (AJCC) stage (*p* < 0.001), pathology (*p* = 0.019), tumor location (*p* < 0.001), extranodal extension (ENE) (*p* < 0.001), systemic immune-inflammation index (SII) (*p* = 0.004), prognostic nutrition index (PNI) (*p* = 0.003), ACCI (*p* < 0.001), and Glasgow prognostic Score (GPS) (*p* = 0.001) were independent indicators for disease free survival (DFS). Additionally, the independent prognostic factors for overall survival (OS) including AJCC stage (*p* = 0.015), pathology (*p* = 0.004), tumor location (*p* < 0.001), perineural invasion (*p* = 0.009), ENE (*p* < 0.001), systemic immune-inflammation index (SII) (*p* = 0.001), PNI (*p* = 0.001), ACCI (*p* = 0.003), and GPS (*p* = 0.033). The nomogram models for predicting DFS and OS in PGC patients were generated based on these independent risk factors. All nomogram models show good discriminative capability with area under curves (AUCs) over 0.8 (DFS 0.802, and OS 0.825, respectively). Decision curve analysis (DCA), integrated discrimination improvement (IDI), and net reclassification index (NRI) show good clinical net benefit of the two nomograms in both training and validation cohorts. Kaplan-Meier survival analyses showed superior discrimination of DFS and OS in the new risk stratification system compared with the AJCC stage system. Finally, postoperative patients with PGC who underwent adjuvant radiotherapy had a better prognosis in the high-, and medium-risk subgroups (*p* < 0.05), but not for the low-risk subgroup.

**Conclusion:**

The immune-inflammatory-nutrition indicators and ACCI played an important role in both DFS and OS of PGC patients. Adjuvant radiotherapy had no benefit in the low-risk subgroup for PGC patients who underwent surgical resection. The newly established nomogram models perform well and can provide an individualized prognostic reference, which may be helpful for patients and surgeons in proper follow-up strategies.

**Supplementary Information:**

The online version contains supplementary material available at 10.1186/s12903-024-04490-5.

## Introduction

Parotid gland carcinoma (PGC) is a rare carcinoma with diverse histologic subtypes, which accounts for less than 3% of head and neck malignancies [1]. The incidence of PGC has been increasing in recent years [2, 3]. According to the Surveillance, Epidemiology, and End Results (SEER) program database, the 3 - and 5-year overall survival (OS) rates for PGC patients were 71.8% and 65.3%, respectively [4]. In the 5th edition of the World Health Organization (WHO) Classification, parotid malignant tumors have been divided into 21 different pathological types [5]. Radical surgical resection is the main treatment for PGC, and some patients also receive adjuvant radiotherapy if necessary [4]. Generally, the location and grade of the PGC are the determining factors for the choice of surgical procedure. The individualized treatment strategy depends on the evaluations of the prognosis of PGC patients [6–8]. At present, the American Joint Committee on Cancer (AJCC) stage system is commonly applied as the main reference for treatment decisions and prognosis prediction in PGC patients [8, 9], which depends on tumor size, anatomical relationship with surrounding structures, lymph node metastasis, and distant metastasis. However, many important prognostic predictors are not included in the AJCC stage, such as age, margin status, pathological type, grade, immune-inflammatory-nutrition indicators, and comorbidities, which may lead to completely different clinical outcomes in clinical practice in PGC patients with the same AJCC stage. Therefore, a more comprehensive and high-efficiency tool with higher predictive power is needed to resolve this clinical problem.

Nomogram is an easily-used and effective tool, which can integrate more prognostic factors to accurately and individually predict the prognosis of patients [10, 11]. At present, many researchers have paid attention to the prognosis prediction of patients with PGC cancer, and many nomogram models have been constructed based on the data from a common database, such as the SEER database. For example, Runqiu Zhu et al. [12] constructed a postoperative nomogram to predict the overall survival (OS) of patients with PGC after surgery based on the SEER database. A competing risk nomogram was developed to predict cancer-specific mortality for patients with PGC by using data from the SEER database [13]. However, there is a lack of nomograms for predicting survival probability in postoperative PGC patients based on local medical data. Moreover, the role of immune-inflammatory-nutrition indicators and Age-Adjusted Charlson Comorbidity Index (ACCI) in predicting the prognosis of PGC patients has not been studied until now.

In this research, we try to figure out the role of immune-inflammatory-nutrition indicators and ACCI in PGC patients and develop prognostic nomograms for estimating the survival probability based on data from local medical centers, which will be helpful in providing personalized guidance.

## Methods

### Cases selection

A total of 344 patients with postoperative PGC from the First Affiliated Hospital of Xinxiang Medical University and the Affiliated Cancer Hospital of Zhengzhou University between March 2012 and June 2018 were enrolled in the study. The inclusion criteria were as follows: (1) pathology has confirmed that it belongs to the primary malignant tumor of the parotid gland, (2) age at diagnosis ≥ 16, and (3) actively followed-up. The exclude criteria include: (1) No surgical resection was performed, (2) surgery status was unknown, (3) 2 or more primary tumors, (4) distant metastasis was present at first diagnosis, (5) accept neoadjuvant chemotherapy or neoadjuvant radiotherapy, (6) pathology undetermined, (7) the necessary clinical data were not available, (8) no follow-up data, and (9) dead within 30 days after surgery. The flow diagram is shown in Supplement. 1. The study was approved by the ethics committees at each institution. Because of the retrospective nature of the study, informed consent was not required.

### Clinicopathological factors

Patients were randomly divided into a training group (241 cases, 70%) and a validation group (103 cases, 30%) by SPSS 20.0. A total of 25 clinicopathological factors of PGC patients were included in the study, including age at diagnosis, gender, consumption of tobacco after surgery, body mass index (BMI), eastern cooperative oncology group (ECOG) performance status (PS) score, pathology, grade, American Joint Committee on Cancer (AJCC) Stage, tumor location, type of resection, vascular invasion (VI), surgical margin, extranodal extension (ENE), perineural invasion, Glasgow prognostic score (GPS), systemic immune-inflammation index (SII), prognostic nutrition index (PNI), platelet-to-lymphocyte ratio (PLR), neutrophil-to-lymphocyte ratio (NLR), hemoglobin (HGB), age-adjusted Charlson comorbidity index (ACCI), bone invasion, adjuvant radiotherapy, disease-free survival (DFS), overall survival (OS). The primary study endpoints were DFS and OS. Intensity-modulated radiotherapy (IMRT) was used as an adjuvant radiotherapy technique. The irradiation dose ranged from 50.0 to 69.9 Gy, once a day, five times per week.

### Calculation

Detailed calculation methods for the ACCI were clearly recorded in Table S1. Similarly, Table S2 shows the calculation formulas of GPS, PNI, NLR, PLR, and BMI.

### Statistical analysis

All statistical analyses were performed with the use of R software, version 4.2.2, and SPSS 20.0. The differences in baseline characteristics between the training and validation groups were compared by the Chi-square test, as well as independent-sample T-test (Table 1). Univariate Cox regression analysis was used to search for variables that had an impact on DFS and OS. The results of the univariate analysis were incorporated into a multivariate Cox regression analysis to identify independent prognostic factors for DFS and OS. *P* < 0.05 was considered the difference was statistically significant. The independent prognostic factors obtained from the multivariate Cox regression analysis were summarized to establish two nomograms for DFS and OS of PGC patients, respectively.

**Table 1:** Clinical information of postoperative patients with parotid gland carcinoma (PGC) in the training and validation groups

Characteristics	All Patients (*n* = 344) *N* (%)	Training cohort (*n* = 241) *N* (%)	Validation cohort (*n* = 103) *N* (%)	*P*
**Age at diagnosis (years)**				0.964
Median (IQR)	50.0 (38-68.75)	50.0 (38–68)	51.0 (38–69)	
**Gender**				0.811
Male	138 (40.1%)	98 (40.7%)	40 (38.8%)	
Female	206 (59.9%)	143 (59.3%)	63 (61.2%)	
**Smoking**				0.06
No	285 (82.8%)	206 (85.5%)	79 (76.7%)	
Yes	59 (17.2%)	35 (14.5%)	24 (23.3%)	
**BMI (kg/m** ^**2**^ **)**				0.954
Median (range)	21.4 (16.0-32.9)	21.4 (16.0-32.9)	21.4 (16.6–31.8)	
**ECOG PS score**				0.771
0–1	264 (76.7%)	186 (77.2%)	78 (75.7%)	
2	80 (23.3%)	55 (22.8%)	25 (24.3%)	
**Pathology**				0.723
Mucoepidermoid carcinoma	225 (65.4%)	157 (65.1%)	68 (66.0%)	
Adenoid cystic carcinoma	50 (14.5%)	38 (15.8%)	12 (11.7%)	
Follicular cell carcinoma	43 (12.5%)	29 (12.0%)	14 (13.6)	
Others^a^	26 (7.6%)	17 (7.1%)	9 (8.7%)	
**Grade**				0.139
I	87 (25.3%)	62 (25.7%)	25 (24.3%)	
II	127 (36.9%)	77 (32.0%)	50 (48.5%)	
III	130 (37.8%)	102 (42.3%)	28 (27.2%)	
**AJCC Stage**				
I	50 (14.5%)	33 (13.7%)	17 (16.5%)	0.673
II	67 (19.5%)	45 (18.7%)	22 (21.4%)	
III	128 (37.2%)	96 (39.8%)	32 (31.1%)	
IVA & IVB	99 (28.8%)	67 (27.8%)	32 (31.1%)	
**Tumor location**				0.524
Superficial	198 (57.6%)	135(56.0%)	63 (61.2%)	
Deep	42 (12.2%)	32 (13.3%)	10 (9.7%)	
Superficial & Deep	104 (30.2%)	74 (30.7%)	30 (29.1%)	
**Type of resection**				0.333
Total parotidectomy	188 (54.7%)	136(56.4%)	52 (50.5%)	
Radical parotidectomy	138 (40.1%)	93 (38.6%)	46 (43.7%)	
Superficial parotidectomy	18 (5.2%)	12 (5.0%)	6 (5.8%)	
**Surgical margin**				0.366
Negative	245 (71.2%)	168 (69.7%)	77 (74.8%)	
Positive	99 (28.8%)	73 (30.3%)	26 (25.2%)	
**VI**				0.350
No	306 (89.0%)	217 (90.0%)	89 (86.4%)	
Yes	38 (11.0%)	24 (10.0%)	14 (13.6%)	
**Perineural invasion**				0.400
No	266 (77.3%)	183 (75.9%)	83 (80.6%)	
Yes	78 (22.7%)	58 (24.1%)	20 (19.4%)	
**ENE**				0.709
Negative	306 (89.0%)	213 (88.4%)	93 (90.3%)	
Positive	38 (11.0%)	28 (11.6%)	10 (9.7%)	
**GPS**				0.557
0	252 (73.3%)	179 (74.3%)	73 (70.9%)	
1	58 (16.9%)	39 (16.2%)	19 (18.4%)	
2	34 (9.9%)	23 (9.5%)	11 (10.7%)	
**SII**				0.190
Median (IQR)	868 (490-1438.5)	904 (491-1477.5)	862 (476–1298)	
**PNI**				0.538
Median (IQR)	70 (54–96)	70 (54–96)	70 (53–92)	
**PLR**				0.580
Median (IQR)	149 (90–202)	149 (90–200)	150 (89–206)	
**NLR**				0.231
Median (IQR)	2.41 (1.32–3.24)	2.33 (1.29–3.20)	2.58 (1.57–3.28)	
**HGB (g/L)**				0.320
Median (IQR)	100 (91–121)	102 (91–123)	98 (91–120)	
**ACCI**				0.105
2–3	144 (41.9%)	105 (43.6%)	39 (37.9%)	
4–5	113 (32.8%)	82 (34.0%)	31 (30.1%)	
≥ 6	87 (25.3%)	54 (22.4%)	33 (32.0%)	
**Bone invasion**				**0.001**
No	271 (78.8%)	205 (85.1%)	66 (64.1%)	
Yes	73 (21.2%)	36 (14.9%)	37 (35.9%)	
**Adjuvant radiotherapy**				0.341
No	214 (62.2%)	146 (60.6%)	68 (66.0%)	
Yes	130 (37.8%)	95 (39.4%)	35 (34.0%)	
**DFS (months)**				0.264
Median (IQR)	34 (13–58)	34 (13-63.5)	32 (12–56)	
**OS (months)**				0.338
Median (IQR)	37 (19-63.75)	38 (19–66)	34 (17–58)	

Others^a^, squamous carcinoma, ductal carcinoma of the salivary gland, papillary cystic carcinoma

*Abbreviations* ACCI, Age-Adjusted Charlson Comorbidity Index; AJCC, American Joint Committee on Cancer; BMI, body mass index; ECOG PS, eastern cooperative oncology group performance status; ENE, extranodal extension; GPS, Glasgow prognostic Score; HGB, hemoglobin; IQR, interquartile range; NLR, neutrophil-to-lymphocyte ratio; PGC, parotid gland carcinoma; PLR, platelet-to-lymphocyte ratio; PNI, prognostic nutrition index; SII, systemic immune-inflammation index; VI, vascular invasion

The concordance index (C-index), the receiver operating characteristic (ROC), the integrated discrimination improvement (IDI), the decision curve analysis (DCA), and the net reclassification improvement (NRI) were calculated by R software to validate the capacity of the nomogram. The C-index and the ROC were used to evaluate the discriminative ability. The consistency between the actual outcome and the predicted probability was evaluated by the calibration curve. Then, the net clinical benefit of the nomograms was evaluated by DCAs. Besides, NRI and IDI were used to compare the predictability of the new model with the AJCC staging system. Finally, according to the risk threshold of the models, the patients in the two cohorts were further divided into high-, medium-, and low-risk groups by X-tile software. The survival time in different risk stratification groups was compared by the log-rank test and Kaplan-Meier plots. In addition, the impact of adjuvant radiotherapy on DFS and OS was analyzed by Log-rank test for the above three subgroups.

## Results

### Clinical characteristics of patients

A total of 344 patients with PGC who underwent surgical resection who meet our criterion were included in this research, and then randomly divided into a training set (*N* = 241) and a validation set (*N* = 103) with a ratio of 7:3 (Supplement. 1), including 206 female patients (59.9%), 285 patients without smoking (82.8%), 225 patients (65.4%) with mucoepidermoid carcinoma and 50 patients (14.5%) with adenoid cystic carcinoma, and 43 patients (12.5%) with follicular cell carcinoma, and 26 (7.6%) patients with others histological types (such as squamous carcinoma, ductal carcinoma of the salivary gland, papillary cystic carcinoma). The median age was 50.0 (IQR: 38-68.75) years old for all patients. Of all cases, 264 patients (76.7%) with ECOG PS scores of 0–1. A total of 198 patients (57.6%) were located at the superficial of parotid. A total of 188 patients (54.7%) underwent total parotidectomy with preservation of facial nerve, 138 patients (40.1%) received radical parotidectomy with sacrifice of facial, and 18 patients (5.2%) with superficial parotidectomy. Most postoperative PGC patients were with negative surgical margins (71.2%) and ENE (89.0%). Moreover, 306 (89.0%) patients without VI, 266 (77.3%) patients without perineural invasion, and 271 (78.8%) patients had no bone invasion. There were 130 (37.8%) patients with PGC who had adjuvant radiotherapy after surgical resection.

There were a series of immune-inflammatory-nutritional indicators selected and analyzed in this study, the indicators of SII (median 868 (IQR: 490-1438.5)), PNI (median 70 (IQR: 54–96)), PLR (median 149 (IQR: 90–202)), NLR (median 2.41 (IQR: 1.32–3.24)) were analyzed as continuous variables. The GPS was analyzed as a categorical variable, which was divided into three groups according to the score of C-reactive protein and albumin, 252 (73.3%) patients with 0 score, 58 (16.9%) patients with 1 score, and 34 (9.9%) patients with 2 score. Moreover, hemoglobin (HGB) is also a marker implicated in nutritional status, which was also included in the analysis with a median of 100 g/l (IQR: 91 g/l-121 g/l).

Besides, ACCI is a marker implicated in comorbidity and age status. A high score of ACCI implies concomitant severe systemic diseases, older age, or other serious conditions, which is suggestive of more conservative treatment modalities and worse prognosis. Most patients in this study had a relatively low score of ACCI, 144 (41.9%) patients with a score of 2–3, 113 (32.8%) patients with a score of 4–5, and 87 (25.3%) with a score of ≥ 6. All baseline clinicopathological characteristics of the two sets were summarized in Table 1. There was no statistical difference in clinicopathological factors except for bone invasion between the patients in the training set and the validation set (*P* > 0.05).

### Construction and validation of prognostic nomogram model for postoperative PGC patients

**Fig. 1:**
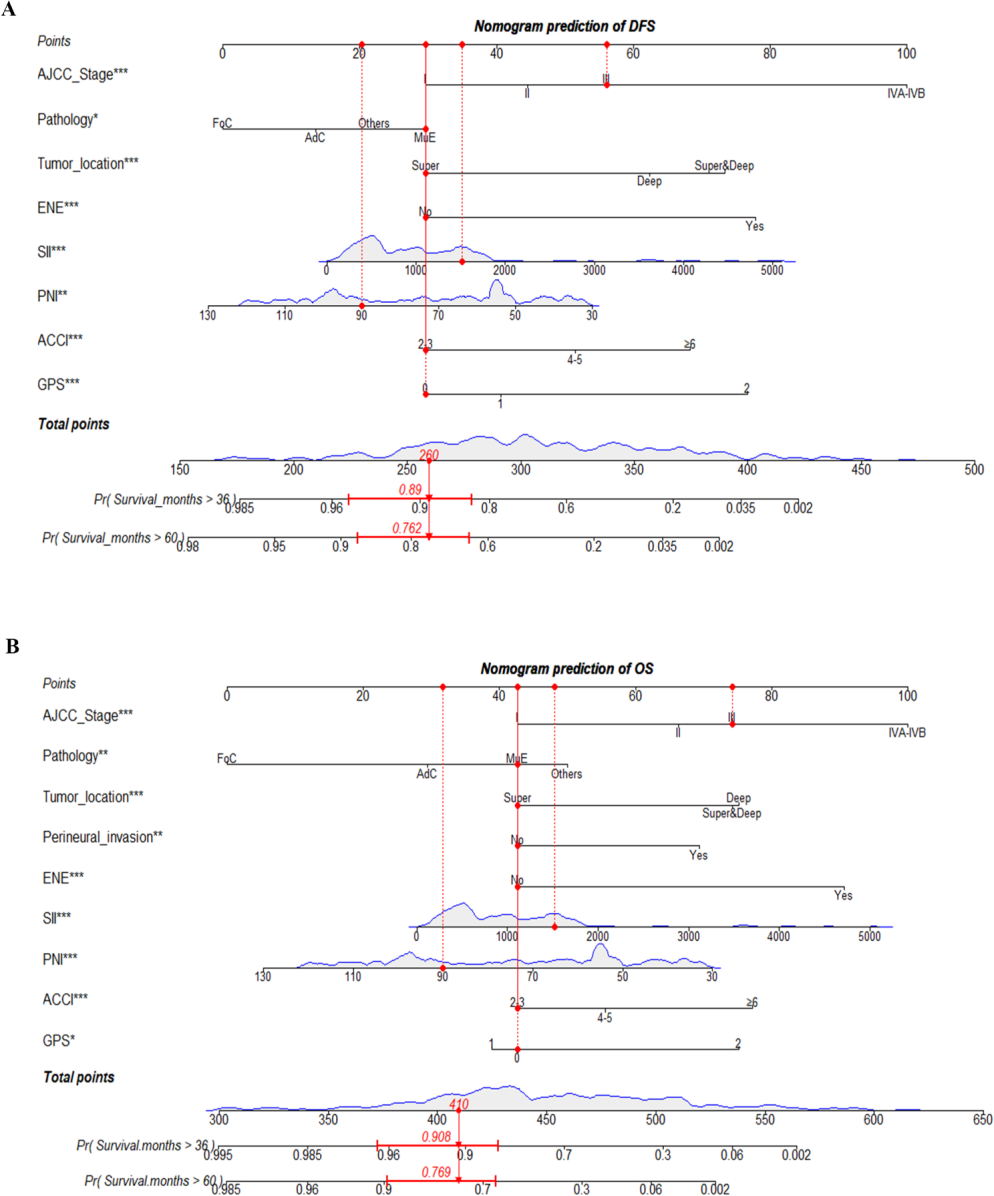
Nomograms to predict 3-, and 5-year disease-free survival (**A**) and overall survival (**B**) for postoperative parotid gland carcinoma (PGC) patients. *P < 0.05, **P < 0.01, ***P < 0.001. ACCI, Age-Adjusted Charlson Comorbidity Index; AJCC, American Joint Committee on Cancer; DFS, disease-free survival; ECOG PS, eastern cooperative oncology group performance status; ENE, extranodal extension; GPS, Glasgow prognostic Score; OS, overall survival; PGC, parotid gland carcinoma; PNI, prognostic nutrition index; SII, Systemic Immune-Inflammation Index

Univariate and multivariate Cox regression analyses were performed to find the predictors for PGC patients who underwent surgical resection in the training group. Eight independent indicators in relation to DFS were identified (Table 2), including AJCC stage (*p* < 0.001), pathology (*p* = 0.019), tumor location (*p* < 0.001), ENE (*p* < 0.001), SII (*p* = 0.004), PNI (*p* = 0.003), ACCI (*p* < 0.001), and GPS (*p* = 0.001). Additionally, we found that patients’ AJCC stage (*p* = 0.015), pathology (*p* = 0.004), tumor location (*p* < 0.001), perineural invasion (*p* = 0.009), ENE (*p* < 0.001), SII (*p* = 0.001), PNI (*p* = 0.001), ACCI (*p* = 0.003), and GPS (*p* = 0.033) were independent predictors of OS in PGC patients (Table 3). Consequently, the nomogram models for predicting DFS (Fig. 1A) and OS (Fig. 1B) in PGC patients were generated based on these independent risk factors found above. The dynamic web-based calculators were also developed in this study, for the prediction of OS: https://xxlchxjh.shinyapps.io/DynNomapp_postoperative_parotid_gland_carcinoma_OS/;for prediction of DFS: https://xxlchxjh.shinyapps.io/DynNomapp_postoperative_parotid_gland_carcinoma_DFS/.

**Table 2:** Univariate and multivariate analyses of clinicopathologic parameters in postoperative patients with parotid gland carcinoma (PGC) for predicting disease-free survival (DFS) in the training group

Characteristics	Univariate analysis		Multivariate analysis	
	HR (95% CI)	*P*	HR (95% CI)	*P*
**Age at diagnosis (years)**	1.013 (1.003–1.023)	**0.014**	0.999 (0.984–1.013)	0.856
**Gender**				
Male	Reference			
Female	1.302 (0.893–1.897)	0.170		
**Smoking**				
No	Reference			
Yes	1.202 (0.761–1.901)	0.430		
**BMI**	0.990 (0.947–1.036)	0.675		
**ECOG PS score**				
0–1	Reference		Reference	
2	1.606 (1.069–2.415)	**0.023**	904 (0.569–1.437)	0.671
**Pathology**				
Mucoepidermoid carcinoma	Reference		Reference	
Adenoid cystic carcinoma	0.555 (0.324–0.950)	**0.032**	0.676 (0.385–1.187)	0.173
Follicular cell carcinoma	0.296 (0.159–0.548)	**< 0.001**	0.446 (0.227–0.878)	**0.019**
Others^a^	0.711 (0.342–1.482)	0.363	0.948 (0.429–2.095)	0.894
**Grade**				
I	Reference			
II	1.283 (0.791–2.081)	0.312		
III	1.458 (0.918–2.314)	0.110		
**AJCC Stage**				
I	Reference		Reference	
II	1.762 (0.818–3.792)	0.148	1.573 (0.691–3.580)	0.280
III	2.585 (1.335–5.004)	**0.005**	1.932 (0.959–3.894)	**0.065**
IVA & IVB	7.562 (3.783–15.115)	**< 0.001**	5.184 (2.471–10.878)	**< 0.001**
**Tumor location**				
Superficial	Reference		Reference	
Deep	2.360 (1.394–3.996)	**0.001**	2.286 (1.307–4.001)	**0.004**
Superficial & Deep	2.071 (1.373–3.122)	**0.001**	2.816 (1.810–4.381)	**< 0.001**
**Type of resection**				
Total parotidectomy	Reference			
Radical parotidectomy	1.034 (0.714–1.498)	0.860		
Superficial parotidectomy	0.353 (0.111–1.121)	0.077		
**Surgical margin**				
Negative	Reference		Reference	
Positive	2.395 (1.642–3.492)	**< 0.001**	1.302 (0.788–2.151)	0.303
**VI**				
No	Reference		Reference	
Yes	1.715 (1.026–2.869)	**0.040**	0.892 (0.502–1.582)	0.695
**Perineural invasion**				
No	Reference		Reference	
Yes	2.584 (1.737–3.843)	**< 0.001**	1.583 (0.984–2.546)	0.058
**ENE**				
Negative	Reference		Reference	
Positive	3.106 (1.913–5.042)	**< 0.001**	2.913 (1.681–5.046)	**< 0.001**
**GPS**				
0	Reference		Reference	
1	1.632 (1.017–2.617)	**0.042**	1.272 (0.762–2.125)	0.358
2	2.328 (1.339–4.048)	**0.003**	2.796 (1.517–5.152)	**0.001**
**SII**	1.000 (1.000-1.001)	**< 0.001**	1.000 (1.000–1.000)	**0.004**
**PNI**	0.987 (0.979–0.994)	**< 0.001**	0.988 (0.979–0.996)	**0.003**
**PLR**	1.001 (0.999–1.003)	0.386		
**NLR**	0.955 (0.813–1.122)	0.576		
**HGB**	0.999 (0.991–1.007)	0.765		
**ACCI**				
2–3	Reference		Reference	
4–5	1.534 (1.006–2.338)	0.047	1.715 (1.100-2.676)	0.017
≥ 6	2.117 (1.339–3.346)	**0.001**	2.574 (1.572–4.216)	**< 0.001**
**Bone invasion**				
No	Reference		Reference	
Yes	7.493 (4.600-12.208)	**< 0.001**	1.779 (0.874–3.621)	0.112
**Adjuvant radiotherapy**				
No	Reference			
Yes	0.727 (0.502–1.053)	0.092		

Others^a^, squamous carcinoma, ductal carcinoma of the salivary gland, papillary cystic carcinoma

*Abbreviations* ACCI, age-adjusted Charlson comorbidity index; AJCC, American Joint Committee on Cancer; BMI, body mass index; CI, confidence interval; DFS, disease-free survival; ECOG PS, eastern cooperative oncology group performance status; ENE, extranodal extension; GPS, Glasgow prognostic Score; HGB, hemoglobin; HR, hazard ratio; NLR, neutrophil-to-lymphocyte ratio; PGC, parotid gland carcinoma; PLR, platelet-to-lymphocyte ratio; PNI, prognostic nutrition index; SII, systemic immune-inflammation index; VI, vascular invasion

**Table 3:** Univariate and multivariate analyses of clinicopathologic parameters in postoperative patients with parotid gland carcinoma (PGC) for predicting overall survival (OS) in the training group

Characteristics	Univariate analysis		Multivariate analysis	
	HR (95% CI)	*P*	HR (95% CI)	*P*
**Age at diagnosis (years)**	1.015 (1.005–1.026)	**0.005**	1.006 (0.991–1.022)	0.425
**Gender**				
Male	Reference			
Female	1.117 (0.750–1.664)	0.587		
**Smoking**				
No	Reference			
Yes	1.183 (0.724–1.932)	0.503		
**BMI**	0.977 (0.931–1.026)	0.351		
**ECOG PS score**				
0–1	Reference			
2	1.429 (0.915–2.231)	0.115		
**Pathology**				
Mucoepidermoid carcinoma	Reference		Reference	
Adenoid cystic carcinoma	0.551 (0.310–0.980)	**0.042**	0.700 (0.383–1.280)	0.247
Follicular cell carcinoma	0.248 (0.124–0.496)	**< 0.001**	0.330 (0.155–0.705)	**0.004**
Others^a^	0.790 (0.362–1.724)	0.554	1.462 (0.618–3.458)	0.387
**Grade**				
I	Reference			
II	1.361 (0.803–2.304)	0.252		
III	1.652 (1.001–2.728)	0.050		
**AJCC Stage**				
I	Reference		Reference	
II	1.718 (0.759–3.891)	0.194	1.950 (0.795–4.783)	0.144
III	2.756 (1.378–5.510)	**0.004**	2.495 (1.158–5.374)	**0.020**
IVA & IVB	7.028 (3.383–14.600)	**< 0.001**	3.199 (1.254–8.161)	**0.015**
**Tumor location**				
Superficial	Reference		Reference	
Deep	2.660 (1.541–4.592)	**< 0.001**	2.164 (1.182–3.961)	**0.012**
Superficial & Deep	1.772 (1.138–2.760)	**0.011**	2.388 (1.475–3.868)	**< 0.001**
**Type of resection**				
Total parotidectomy	Reference			
Radical parotidectomy	0.956 (0.642–1.424)	0.824		
Superficial parotidectomy	0.416 (0.130–1.328)	0.139		
**Surgical margin**				
Negative	Reference		Reference	
Positive	2.386 (1.596–3.568)	**< 0.001**	1.001 (0.563–1.782)	0.996
**VI**				
No	Reference		Reference	
Yes	1.821 (1.053–3.149)	**0.032**	0.817 (0.432–1.544)	0.534
**Perineural invasion**				
No	Reference		Reference	
Yes	2.836 (1.872–4.296)	**< 0.001**	1.978 (1.187–3.296)	**0.009**
**ENE**				
Negative	Reference		Reference	
Positive	3.977 (2.428–6.515)	**< 0.001**	3.008 (1.767–5.391)	**< 0.001**
**GPS**				
0	Reference		Reference	
1	1.359 (0.812–2.277)	0.243	0.900 (0.513–1.577)	0.712
2	2.159 (1.169–3.989)	**0.014**	2.120 (1.064–4.221)	**0.033**
**SII**	1.000 (1.000-1.001)	**< 0.001**	1.000 (1.000-1.001)	**0.001**
**PNI**	0.984 (0.976–0.992)	**< 0.001**	0.984 (0.975–0.993)	**0.001**
**PLR**	1.001 (0.999–1.003)	0.180		
**NLR**	0.978 (0.823–1.162)	0.798		
**HGB**	0.998 (0.989–1.006)	0.622		
**ACCI**				
2–3	Reference		Reference	
4–5	1.438 (0.919–2.250)	0.112	1.242 (0.761–2.026)	0.386
≥ 6	2.027 (1.241–3.311)	**< 0.001**	2.260 (1.329–3.843)	**0.003**
**Bone invasion**				
No	Reference		Reference	
Yes	7.697 (4.716–12.563)	**< 0.001**	2.233 (0.979–5.093)	0.056
**Adjuvant radiotherapy**				
No	Reference			
Yes	0.698 (0.467–1.042)	0.078		

Others^a^, squamous carcinoma, ductal carcinoma of the salivary gland, papillary cystic carcinoma

*Abbreviations* ACCI, age-adjusted Charlson comorbidity index; AJCC, American Joint Committee on Cancer; BMI, body mass index; CI, confidence interval; ECOG PS, eastern cooperative oncology group performance status; ENE, extranodal extension; GPS, Glasgow prognostic Score; HGB, hemoglobin; HR, hazard ratio; NLR, neutrophil-to-lymphocyte ratio; OS, overall survival; PGC, parotid gland carcinoma; PLR, platelet-to-lymphocyte ratio; PNI, prognostic nutrition index; SII, systemic immune-inflammation index; VI, vascular invasion

**Fig. 2:**
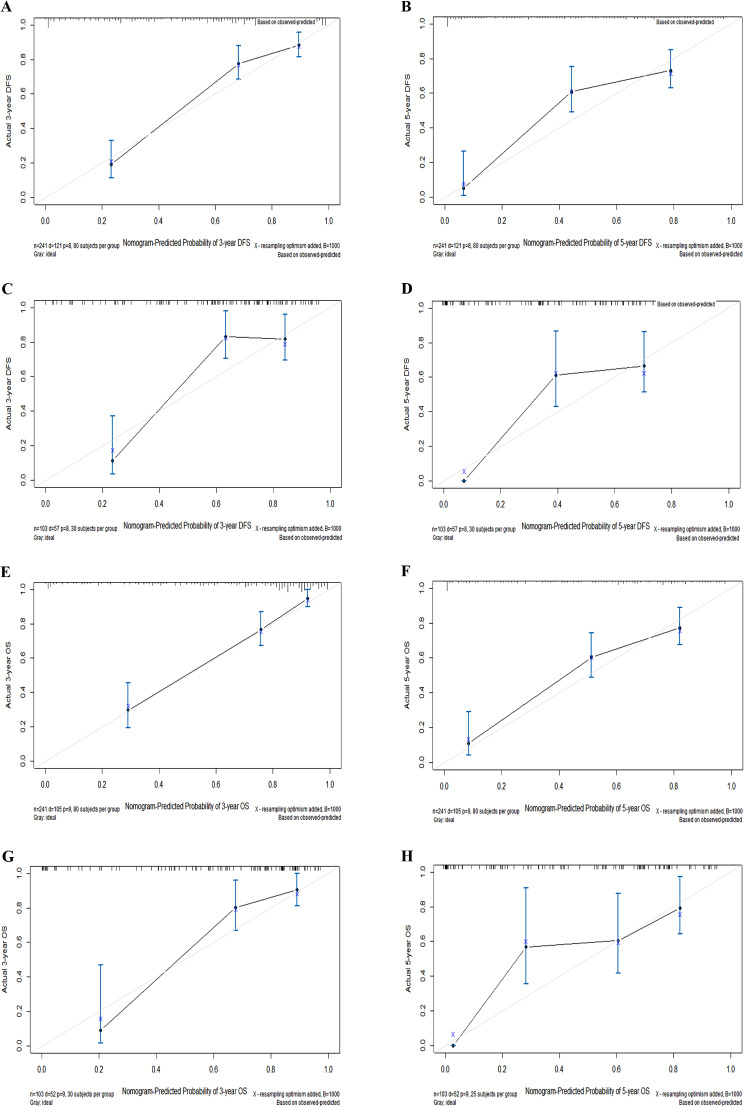
Calibration plots of 3-, and 5-year DFS (**A**-**D**) and OS (**E**-**H**) for postoperative parotid gland carcinoma (PGC). (**A**, **B**) Calibration plots of 3-, and 5-year DFS in the training cohort. (**C**, **D**) Calibration plots of 3-, and 5-year DFS in the validation cohort. (**E**, **F**) Calibration plots of 3-, and 5-year OS in the training cohort. (**G**, **H**) Calibration plots of 3-, and 5-year OS in the validation cohort. OS, overall survival; DFS, disease-free survival

Calibration plots and time-dependent ROC curves show good predictive performance of these nomogram models for predicting DFS and OS. Calibration plots showed a high degree of consistency between the predicted survival and the actual survival in both training and validation cohorts (Fig. 2). Moreover, the AUCs for predicting DFS at 3-, and 5-year in the training cohort were 0.891, and 0.853, respectively (Supplement. 2 A), and those were 0.847, and 0.845 in the validation cohort, respectively (Supplement. 2B). The AUCs at 3-, and 5-year for predicting OS were 0.891, and 0.862 in the training set, respectively (Supplement. 2 C), and those were 0.897, and 0.857 in the validation set, respectively (Supplement. 2D). All these results demonstrated that the two nomogram models show good discriminative capability as all AUCs were over 0.8. The C-index of the nomogram for predicting DFS and OS were 0.802, and 0.825 in the training group, respectively, and those were 0.783, and 0.839 in the validation group (Table 4). All C-index for newly established nomograms were higher than the traditional AJCC stage system.

**Fig. 3:**
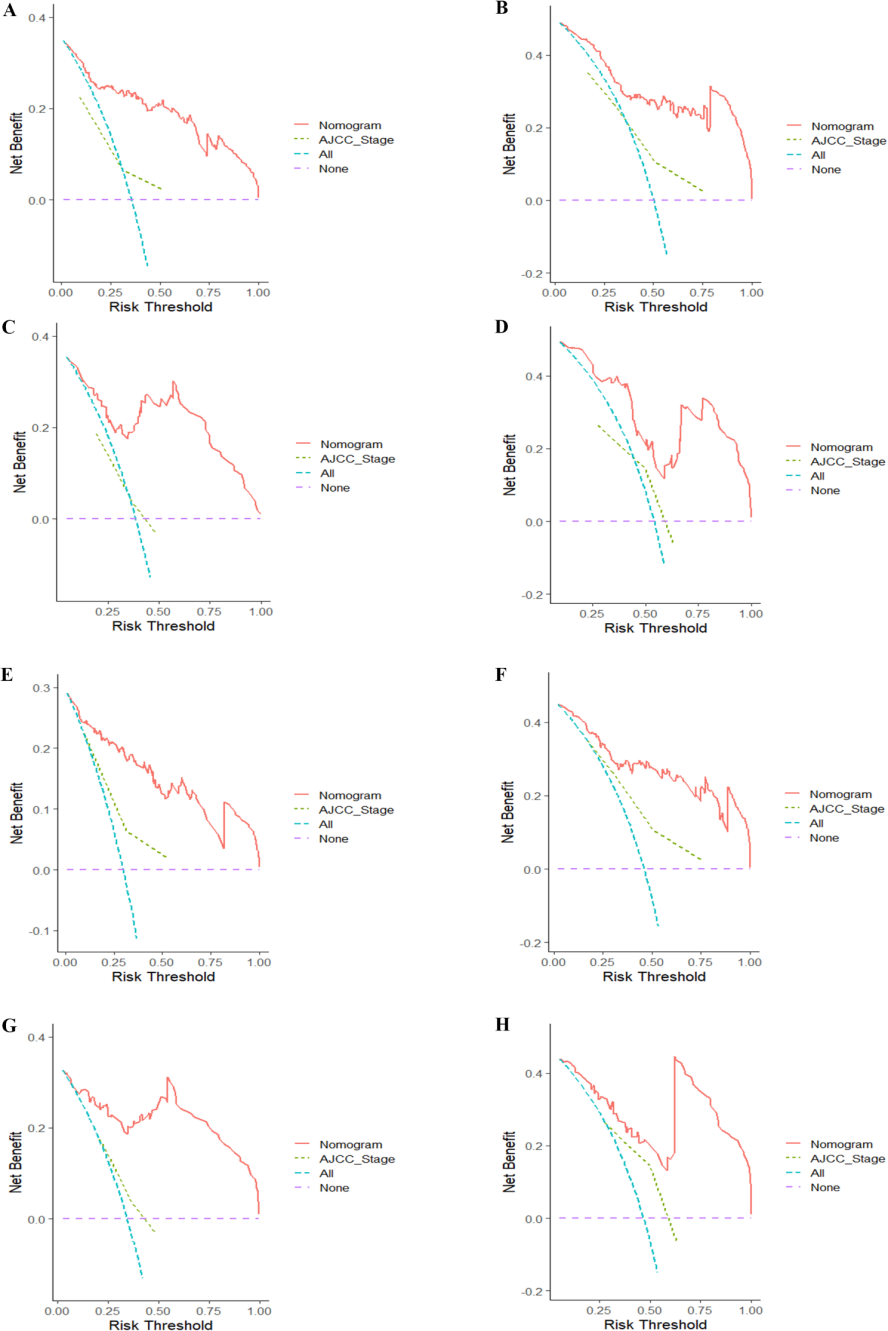
Decision curve analysis of the DFS-associated and OS-associated nomograms. DCA curves of 3-, and 5-year DFS in the training cohort (**A**, **B**) and validation cohort (**C**, **D**). DCA curves of 3-, and 5-year OS in the training group (**E**, **F**) and validation group (**G**, **H**)

The superiority of the newly generated nomograms based on immune-inflammatory indicators and ACCI was evaluated by DCA, IDI, and NRI. It is obvious that the clinical net benefit of newly established nomograms was better than the AJCC stage system in both the training and validation cohort (Fig. 3). Moreover, The NRI at 3-, and 5-year were 0.534, and 0.481 for DFS, respectively, and those were 0.545, and 0.512 for OS in the training set, respectively. In the validation set, the NRI were 0.542, and 0.529 for DFS, respectively, and those were 0.688, and 0.578 for OS at 3-, and 5-year, respectively (Table 4). The IDI for 3-, and 5-year DFS in the training group were 0.244, and 0.481, respectively, and those were 0.290, and 0.240 in the validation group. For the prediction of 3-, and 5-year OS, the IDI was 0.279, and 0.244 in the training group, respectively, and those were 0.424, and 0.337 in the validation group, respectively (Table 4).

**Table 4:** The IDI, NRI, and C-index of the nomograms and AJCC Stage system in OS and DFS prediction for PGC patients after surgery

Index	Training cohort		*P*	Validation cohort		*P*
	Estimate	95%CI		Estimate	95%CI	
NRI (vs. AJCC Stage system)						
For 3-year OS	0.545	0.358–0.698		0.688	0.443–0.843	
For 5-year OS	0.512	0.295–0.638		0.578	0.316–0.783	
For 3-year DFS	0.534	0.335–0.652		0.542	0.281–0.750	
For 5-year DFS	0.481	0.325–0.631		0.529	0.198–0.716	
**IDI (vs. AJCC** **Stage system)**						
For 3-year OS	0.279	0.192–0.389	**< 0.001**	0.424	0.290–0.556	**< 0.001**
For 5-year OS	0.244	0.128–0.349	**< 0.001**	0.337	0.203–0.465	**< 0.001**
For 3-year DFS	0.244	0.138–0.342	**< 0.001**	0.290	0.193–0.426	**< 0.001**
For 5-year DFS	0.481	0.325–0.631	**< 0.001**	0.240	0.136–0.368	**< 0.001**
**C-index**						
The nomogram (OS)	0.825	0.790–0.860		0.839	0.790–0.888	
The nomogram (DFS)	0.802	0.765–0.839		0.783	0.730–0.836	
The AJCC Stage (OS)	0.688	0.637–0.739		0.656	0.757 − 0.738	
The AJCC Stage (DFS)	0.689	0.642–0.736		0.670	0.592–0.748	

*Abbreviations* AJCC, American joint committee on cancer; CI, confidence interval; C-index, concordance index; DFS, disease-free survival; IDI, integrated discrimination improvement; NRI, net reclassification index; OS, overall survival; PGC, parotid gland cancer

### Risk stratification system based on the nomogram

**Fig. 4:**
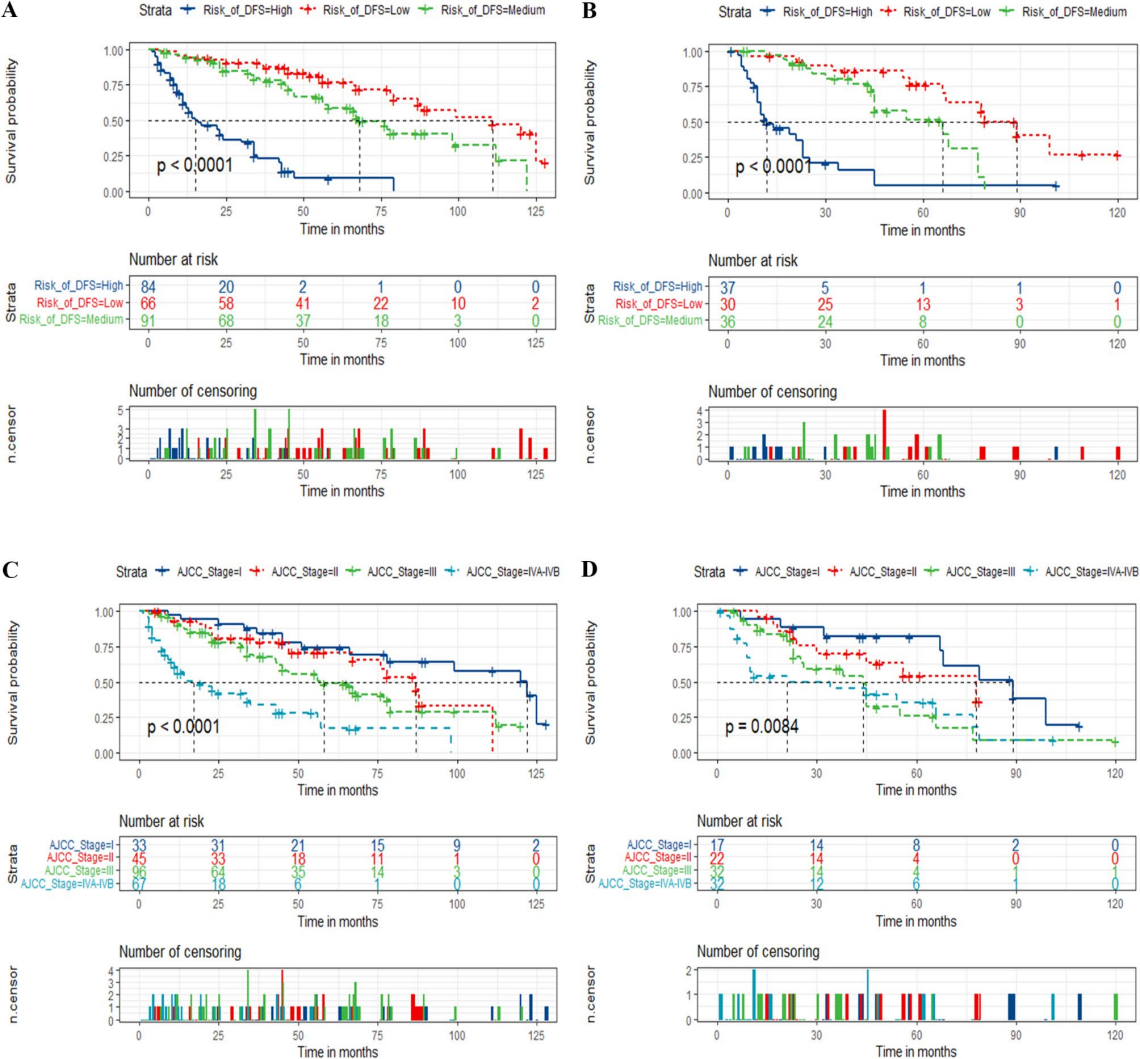
Kaplan–Meier curves of postoperative patients with PGC for predicting DFS based on the new risk stratification system and the AJCC stage system. (**A**, **B**) Kaplan–Meier curves in the training (**A**) and validation cohorts (**B**) according to the new risk stratification system. (**C**, **D**) Kaplan–Meier curves according to the AJCC Stage system of the training (**C**) and validation cohorts (**D**)

**Fig. 5:**
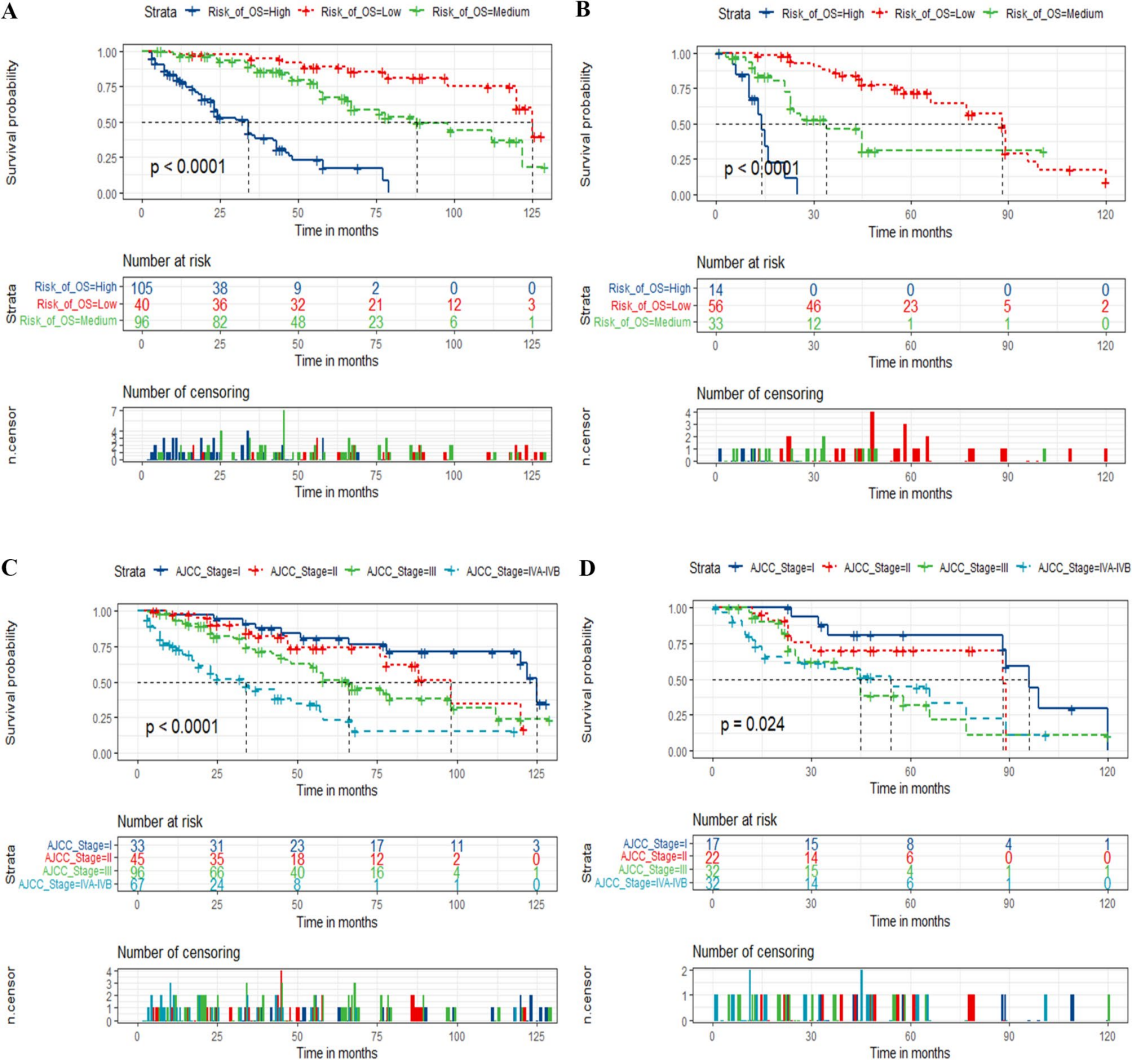
Kaplan–Meier curves of postoperative patients with PGC for predicting OS based on the new risk stratification system and the AJCC stage system. (**A**, **B**) Kaplan–Meier curves in the training (**A**) and validation cohorts (**B**) according to the new risk stratification system. (**C**, **D**) Kaplan–Meier curves according to the AJCC Stage system of the training (**C**) and validation cohorts (**D**)

**Fig. 6:**
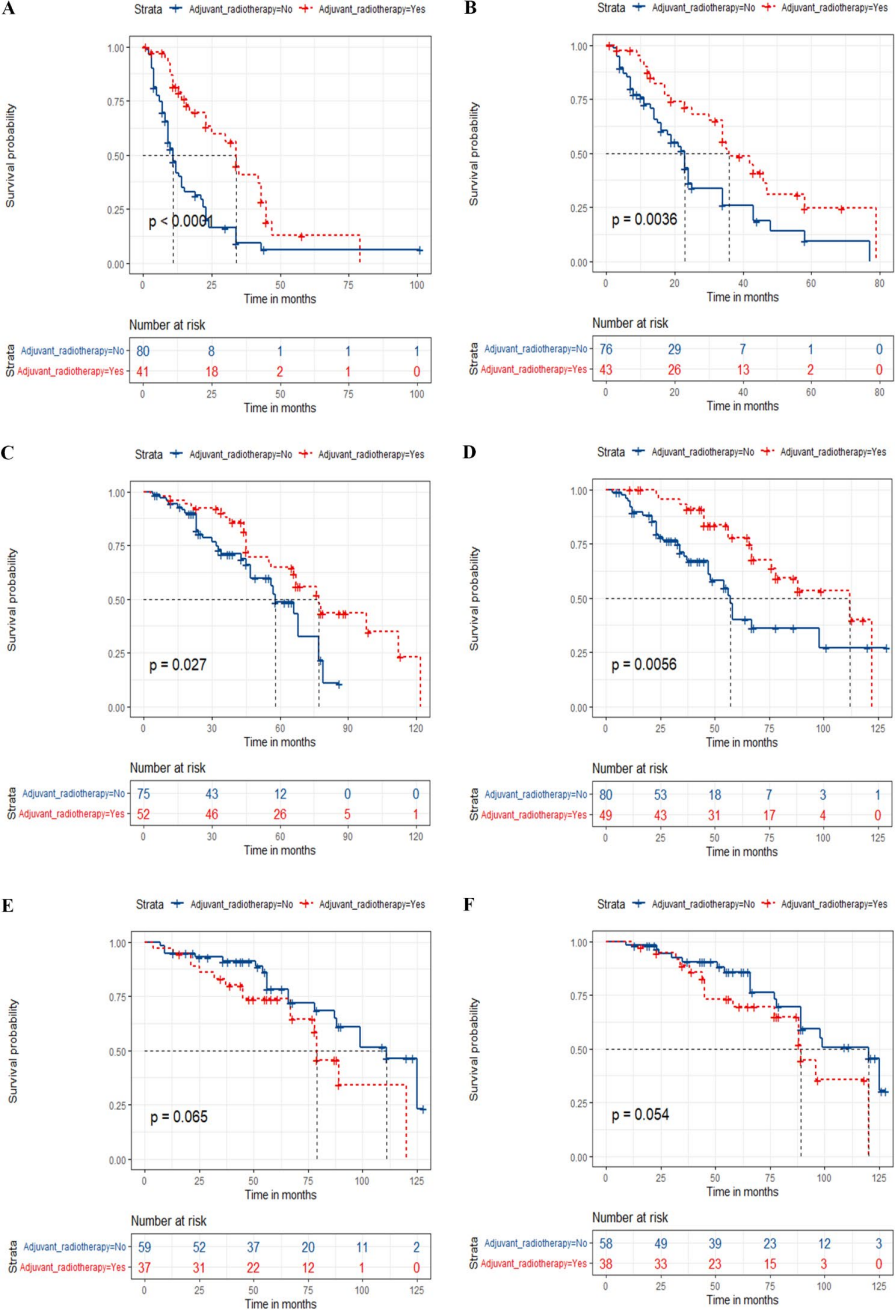
Kaplan–Meier curves for predicting DFS (**A**, **C**, **E**) and OS (**B**, **D**, **F**) based on the new risk stratification system of all postoperative parotid gland carcinoma (PGC) patients. Kaplan–Meier curves of patients with or without adjuvant radiotherapy in the high- (**A**), medium- (**C**), and low-risk group (**E**) for predicting DFS; Kaplan–Meier OS curves of patients with or without adjuvant radiotherapy for predicting OS in the high- (**B**), medium-(**D**), and low-risk group (**F**)

According to the total score, the risk stratification system was established by using X-tile software. Patients were then separated into three risk cohorts. The optimal cut-off points were low- (≤ 160.43), medium- (162.15-225.44), and high-risk (≥ 225.5) for prediction of DFS, respectively, and those were low- (≤ 124.92), medium- (124.92-183.47), and high-risk (≥ 183.70) for prediction of OS, respectively. Kaplan-Meier survival analyses showed that the DFS and OS of patients in the new risk stratification system displayed superior discrimination in comparison with the AJCC stage system (Figs. 4 and 5). The high-risk subgroup had a worse prognosis than those in medium- and low-risk subgroups both in the training and validation groups (*p* < 0.001). Additionally, postoperative patients with PGC in low-risk subgroup could not get a better prognosis from adjuvant radiotherapy (Fig. 6), which means that postoperative adjuvant radiotherapy does not improve the prognosis of all PGC patients (*p* > 0.05).

## Discussion

PGC is a rare head and neck tumor with remarkable heterogeneity, which results highly variable in treatment outcomes. Generally, the AJCC stage system was the widely-used prognostic tool for PGC, which is mainly based on tumor size, lymph node status, and distant metastasis. Other important patient- or disease-related variables were not included and considered, such as age, ECOG PS, tumor grade, tumor location, surgical margin, perineural invasion, VI, ENE, immune-inflammatory-nutritional indicators, ACCI, etc. Therefore, it is important to identify more prognostic factors and develop a comprehensive and applicable tool to upregulate the efficiency in predicting prognosis, which can be helpful for clinicians in making proper clinical decisions.

PGC has been studied previously by various researchers from different aspects [4, 6, 12, 14, 15]. However, the role of immune-inflammatory-nutrition indicators and Age-Adjusted Charlson Comorbidity Index (ACCI) in PGC patients has not been studied so far. In this study, we obtained the total cases from two tertiary hospitals in China and focused on the role of immune-inflammatory-nutrition indicators and ACCI in PGC patients which had not been noticed before. Based on our results, a series of immune-inflammatory-nutrition indicators (SII, PNI, PLR, NLR, GPS), and ACCI were important prognostic factors for predicting OS and DFS. Two nomograms for predicting OS and DFS were constructed according to the identified independent predictors, which can be easily acquired from postoperative PGC patients in clinical practice. Additionally, adjuvant radiotherapy was found to be beneficial for high-, and medium-risk postoperative PGC patients except for those in the low-risk subgroup. Consequently, user-friendly nomograms for estimating 3- and 5-year OS and DFS after surgical resection in PGC patients were established by personalized clinical parameters.

Recently, the understanding of the immune-inflammatory microenvironment and nutritional status has gradually deepened with the development of modern medicine. These immune-inflammatory-nutrition-related factors have been found to play an important role in tumor proliferation, invasion, immune escape, treatment tolerability, response to treatment, etc [16–19]. However, the relationship between immune-inflammatory-nutrition-related factors and the prognosis of PGC had not been studied previously. In this study, we pay close attention to the aspects and found that high SII, PNI, and GPS indicate a worse prognosis for the first time, which is consistent with the role of the immune-inflammatory-nutrition-related factors in other types of cancer. These findings suggest that the immune-inflammatory and nutritional status should be concerned for postoperative PGC patients, which is an important reference for surgeons and patients.

The presence or absence of complications is one of the important indicators to evaluate surgical indications. ACCI, a comprehensive assessment of comorbidities and age, has been reported to predict survival time in a variety of tumors [20, 21]. The correlation between ACCI and PGC has not been studied until now. In our research, the ACCI was been brought into the analysis for the first time and displayed an important role in the prognosis prediction of PGC patients with surgical resection. It is obvious that the higher the ACCI score, the worse the OS and DFS of PGC patients. The index of ACCI is another important supplement to accurately evaluate the prognosis of PGC patients.

The perineural invasion and ENE were reported as important prognostic factors in head and neck cancer [22–24], which were vital evaluation indicators of postoperative patients. In our study, most postoperative PGC patients presented without perineural invasion and ENE. The survival analysis indicates that positive perineural invasion and ENE were prone to worse OS and DFS in PGC patients with surgical resection, which is consistent with the previous studies.

Other important frequently-used clinicopathological factors including tumor location and pathology were also been found to play an important role in the prognosis prediction of PGC patients in this research. Besides, we also found that postoperative PGC patients in the low-risk subgroup could not benefit from adjuvant radiotherapy, which is a vital supplement for the current standard of indications for postoperative radiotherapy. The newly comprehensive nomogram models and risk stratification systems were constructed based on these results, which make up shortcomings for the existing AJCC stage system and related standards to a certain extent, and are helpful for accurately evaluating the prognosis of individuals with PGC underwent surgical resection, as well as provide a reference for individualized treatment.

There are still some limitations in this research. Firstly, the sample size is limited which should be expanded in the future. Secondly, there is an inevitable selection bias in the retrospective study. Thirdly, other factors that may also have an influence on the prognosis of PGC were not included in this study, such as socioeconomic status, drinking, and so on.

## Conclusion

A series of independent prognostic factors for OS and DFS in postoperative PGC patients were identified in this study, including immune-inflammatory-nutrition indicators, ACCI, ENE, tumor location, pathology, AJCC stage, and perineural invasion. The two nomograms were developed based on those predictors, which showed good performance and clinical benefit. The new risk stratification system accurately distinguishes low-, medium- and high-risk subgroups. Besides, adjuvant radiotherapy had no benefit for low-risk subgroups of PGC patients with surgical resection.

### Electronic supplementary material

Below is the link to the electronic supplementary material.

Supplementary Material 1

Supplementary Material 2

Supplementary Material 3

Supplementary Material 4

## Data Availability

Detailed data are available from the corresponding author upon reasonable request.

## Abbreviations

ACCI: Age-Adjusted Charlson Comorbidity Index
AJCC: American Joint Committee on Cancer
AUC: area under curve
BMI: body mass index
CI: confidence interval
C-index: concordance index
DCA: decision curve analysis
DFS: disease-free survival
ECOG PS: eastern cooperative oncology group performance status
ENE: extranodal extension
GPS: Glasgow prognostic Score
HGB: hemoglobin
IDI: integrated discrimination improvement
IMRT: intensity-modulated radiotherapy
IQR: interquartile range
NLR: neutrophil-to-lymphocyte ratio
OS: overall survival
PGC: parotid gland carcinoma
PLR: platelet-to-lymphocyte ratio
PNI: prognostic nutrition index
ROC: receiver operating characteristic
SEER: Surveillance: Epidemiology: and End Results
SII: systemic immune-inflammation index
VI: vascular invasion
WHO: World Health Organization
