# Supplementary figures and images for: Nomogram based on immune-inflammatory indicators and age-adjusted charlson comorbidity index score to predict prognosis of postoperative parotid gland carcinoma patients
Source: BMC Oral Health. 2024 Jun 22;24:718. doi: 10.1186/s12903-024-04490-5 (PMC11193213; doi:10.1186/s12903-024-04490-5)

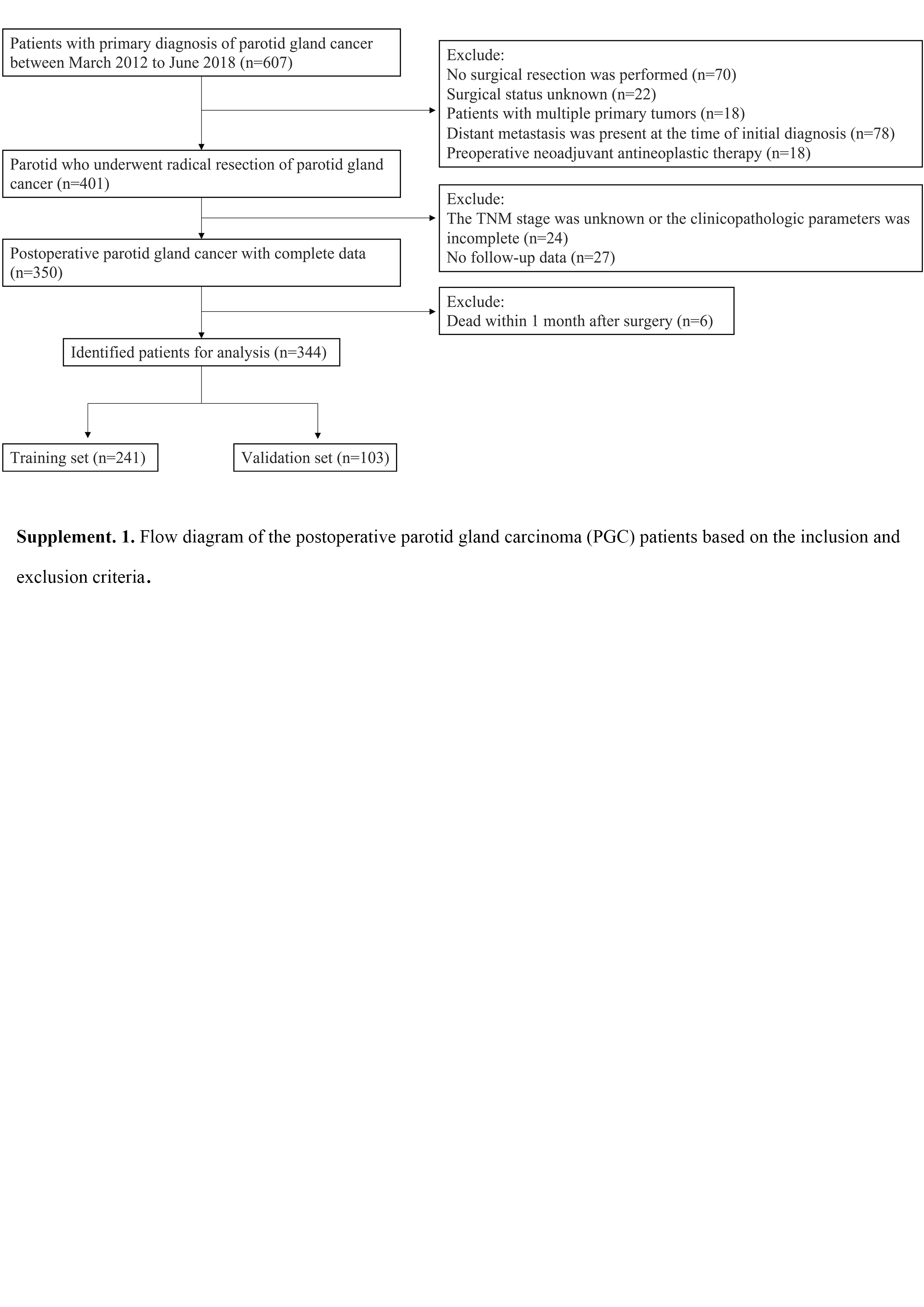

Supplement: Supplementary file 1 — Supplementary Material 1 [file 12903_2024_4490_MOESM1_ESM.tif]

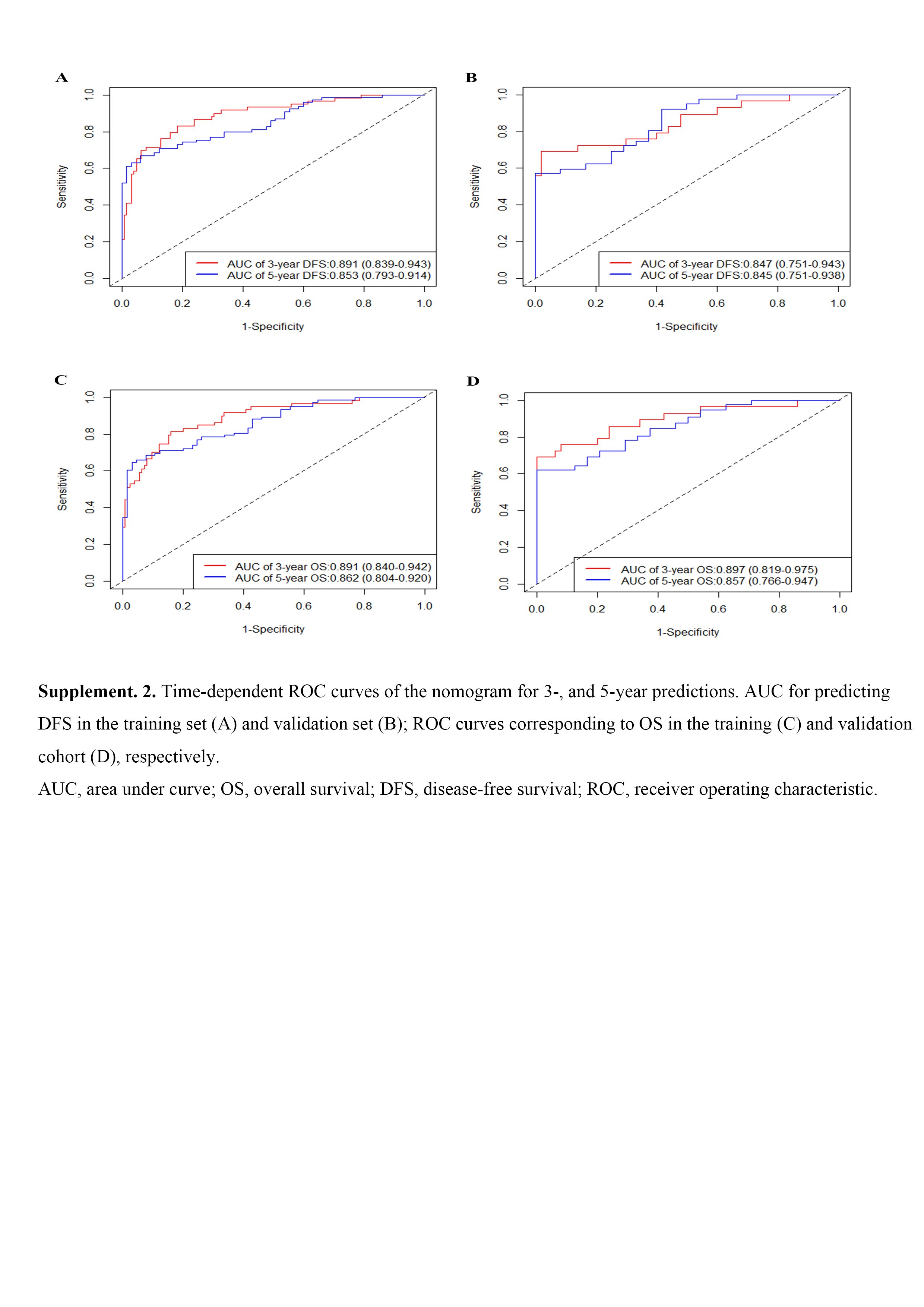

Supplement: Supplementary file 2 — Supplementary Material 2 [file 12903_2024_4490_MOESM2_ESM.tif]
